# Small non-coding RNAs: from trash to treasure

**DOI:** 10.1590/2175-8239-JBN-2019-0075

**Published:** 2019-07-10

**Authors:** Cristian V. Riella

**Affiliations:** 1 Harvard Medical School Beth Israel Deaconess Medical Center BostonMassachusetts United States Harvard Medical School, Beth Israel Deaconess Medical Center, Boston, Massachusetts, United States.

Since the discovery of the first microRNA in 1993, our non-coding RNA knowledge has grown exponentially^1^. Previously thought as transcriptional trash, given the lack of translation into protein, non-coding RNAs are now known to play critical biological roles^2^^-^4 A number of different types of non-coding RNA have been discovered, the most prominent ones include long non-coding RNAs, microRNAs (miRNA), and small interfering RNAs (siRNA). The first miRNA discovered was described in the nematode *C. elegans*, and twenty-six years later there are over 2600 (miRBase V22) miRNAs annotated to the human genome^5^

MiRNA molecules are 21-22 nucleotides long and bind to proteins in order to form complexes (RNA-induced silencing complex, or RISC) to then target messenger RNA transcripts with base-pair complementarity to the miRNA. The targeted messenger RNA may have its translation blocked or its transcript degraded. The advent of next-generation sequencing has potentiated the discovery of new miRNAs at a much faster rate. Two decades after the discovery, we are starting to scratch the surface of the possibilities of miRNAs' use as biomarkers, diagnositc and prognostic tools.

In this edition of the BJN, Milhoransa P et al.^6^ assessed the expression of miR-146a-5p in 55 kidney transplant recipients and compared stable graft function controls (SGF) with delayed graft function (DGF) and acute rejection (AR) individuals. The authors demonstrated that miR-146a-5p was differentially expressed in DGF kidney biopsy samples versus SGF (DGF median=3.23; interquartile [IQR]=1.46-5.74 vs. SGF median=0.78; IQR=0.57-1.99; P=0.019). Both AR and SGF groups had lower levels of miR-146a-5p. The peripheral blood quantification of miR-146a-5p did not demonstrate differential expression in the DFG group vs. AR and SGF groups. Another important finding was that peripheral blood expression levels of miR-146a-5p did not correlate with the kidney tissue expression levels.

The authors hypothesized that miR-146a-5p would have higher expression in DGF patients and potentially serve as a non-invasive marker. The hypothesis is based on previous studies on experimental mouse models of ischemia-reperfusion injury and IgA nephropathy in which higher levels correlated with the degree of injury. The miR-146a-5p is thought to play a central role in innate immune response, specifically in immune cells, but also in kidney cells. The current article confirmed the hypothesis in kidney tissue of DGF patients.

MiRNAs are an attractive molecule for the development of a non-invasive biomarker. They are present intracellularly but also remain stable in blood and urine due to protein binding and inside exosomes, therefore resisting enzymatic degradation through ribonucleases.^7^ This study presented a molecule with good correlation between DGF outcomes and expression levels. More work is needed to make it ready for prime time given the specificity and sensitivity in the 60-70% range. The vast number of different miRNAs acting on each cell simultaneously poses a tough challenge to identify a single miRNA with the strongest association to the outcome of interest. Besides, miRNAs are tissue-specific, having different functions and expression patterns in different cell types depending on stimuli.

The presented findings are limited by the study’s low number of subjects, especially in the control group (n=13), which showed a high interquartile variation for miR-146a-5p expression, therefore hindering the statistical power. As the authors proposed, a targeted and cost-effective way to assess the kidney compartment would be to isolate miRNAs from urine, which has been proven to be reliable and practical.^7^

The transplant field is gasping for a non-invasive allograft function marker that is low-cost, reproducible, and able to detect allograft dysfunction early, for a timely intervention and consequently improved long-term outcomes. The authors presented exciting new findings that with optimization will bring the assay closer to a suitable biomarker. More than one miRNA might be necessary to achieve the sensitivity and specificity required for a screening test. For now, we have to settle for creatinine, a test that detects kidney injury a few days too late.
